# Phytoactive-Loaded Lipid Nanocarriers for Simvastatin Delivery: A Drug Repositioning Strategy Against Lung Cancer

**DOI:** 10.3390/pharmaceutics17020255

**Published:** 2025-02-14

**Authors:** Rocío Gambaro, Cecilia Y. Chain, Sebastian Scioli-Montoto, Ailin Moreno, Cristián Huck-Iriart, María Esperanza Ruiz, José S. Cisneros, Diego G. Lamas, Julia Tau, Stephan Gehring, Germán A. Islan, Boris Rodenak-Kladniew

**Affiliations:** 1 Children’s Hospital, University Medical Center of the Johannes, Gutenberg University, Langenbeckstr. 1, 55131 Mainz, Germany; gambaror@uni-mainz.de (R.G.); stephan.gehring@uni-mainz.de (S.G.); 2 Instituto de Investigaciones Fisicoquímicas Teóricas y Aplicadas (INIFTA), Consejo Nacional de Investigaciones Científicas y Tecnológicas (CONICET)-Universidad Nacional de La Plata (UNLP), La Plata 1900, Buenos Aires, Argentina; yamil@inifta.unlp.edu.ar (C.Y.C.); josesebastiancisneros@biol.unlp.edu.ar (J.S.C.); 3 Laboratorio de Investigación y Desarrollo de Bioactivos (LIDeB), Departamento de Ciencias Biológicas, Facultad de Ciencias Exactas, Universidad Nacional de La Plata, La Plata 1900, Buenos Aires, Argentina; scioli.montoto@biol.unlp.edu.ar (S.S.-M.); eruiz@biol.unlp.edu.ar (M.E.R.); 4 Instituto de Investigaciones Bioquímicas de La Plata (INIBIOLP), Investigaciones Científicas y Tecnológicas (CONICET)-Universidad Nacional de La Plata (UNLP), CCT-La Plata, Facultad de Ciencias Médicas UNLP, La Plata 1900, Buenos Aires, Argentina; aimoreno@uade.edu.ar (A.M.); citometro@med.unlp.edu.ar (J.T.); 5 Instituto de Tecnologías Emergentes y Ciencias Aplicadas (ITECA), Universidad Nacional de San Martín (UNSAM)--Investigaciones Científicas y Tecnológicas (CONICET), Escuela de Ciencia y Tecnología (ECyT), Laboratorio de Cristalografía Aplicada (LCA), Campus Miguelete, San Martín 1650, Buenos Aires, Argentina; chuck@unsam.edu.ar (C.H.-I.); dlamas@unsam.edu.ar (D.G.L.); 6 ALBA Synchrotron Light Source, Carrer de la Llum 2–26, Cerdanyola del Vallès, 08290 Barcelona, Spain; 7 Centro de Investigación y Desarrollo en Fermentaciones Industriales (CINDEFI), Laboratorio de Nanobiomateriales, Departamento de Química, Facultad de Ciencias Exactas, Investigaciones Científicas y Tecnológicas (CONICET)-Universidad Nacional de La Plata (UNLP), CCT-La Plata, La Plata 1900, Buenos Aires, Argentina

**Keywords:** plant-derived monoterpenes, simvastatin, bioactive lipid nanoparticles, synergism, drug repositioning, lung cancer, anticancer mechanisms

## Abstract

**Background/Objectives:** Drug repurposing explores new applications for approved medications, such as simvastatin (SV), a lipid-lowering drug that has shown anticancer potential but is limited by solubility and side effects. This study aims to enhance SV delivery and efficacy against lung cancer cells using bioactive lipid nanoparticles formulated with plant-derived monoterpenes as both nanostructuring agents and anticancer molecules. **Methods:** Lipid nanoparticles were produced by ultrasonication and characterized for morphology, size, zeta potential, and polydispersity index (PDI). Monoterpenes (linalool-LN-, limonene, 1,8-cineole) or Crodamol^®^ were used as liquid lipids. Encapsulation efficiency (EE), release profiles, stability, biocompatibility, protein adsorption, cytotoxicity, and anticancer effects were evaluated. **Results:** The nanoparticles exhibited high stability, size: 94.2 ± 0.9–144.0 ± 2.6 nm, PDI < 0.3, and zeta potential: −4.5 ± 0.7 to −16.3 ± 0.8 mV. Encapsulation of SV in all formulations enhanced cytotoxicity against A549 lung cancer cells, with NLC/LN/SV showing the highest activity and being chosen for further investigation. Sustained SV release over 72 h and EE > 95% was observed for NLC/LN/SV. SAXS/WAXS analysis revealed that LN altered the crystallographic structure of nanoparticles. NLC/LN/SV demonstrated excellent biocompatibility and developed a thin serum protein corona in vitro. Cellular studies showed efficient uptake by A549 cells, G0/G1 arrest, mitochondrial hyperpolarization, reactive oxygen species production, and enhanced cell death compared to free SV. NLC/LN/SV more effectively inhibited cancer cell migration than free SV. **Conclusions:** NLC/LN/SV represents a promising nanocarrier for SV repurposing, combining enhanced anticancer activity, biocompatibility, and sustained stability for potential lung cancer therapy.

## 1. Introduction

Lung cancer remains the main cause of cancer-related deaths worldwide, comprising 11.4% of all cancer diagnoses and 18.0% of cancer-associated deaths in 2020, with a mortality rate of 1.79 million [1]. It is primarily classified into two types: non-small cell lung cancer (NSCLC), accounting for 85% of cases, and small cell lung cancer (SCLC). Lung cancer exhibits high mortality and incidence rates, exceeding 80%, and has a 5-year survival rate of only 10–20% [1]. Despite the emergence of promising therapies such as immunotherapy and targeted drug therapy, challenges such as drug resistance, adverse effects, and high economic costs have contributed to persistently low survival rates in lung cancer patients [2,3,4]. In this context, chemotherapy remains central to lung cancer treatment [5,6], despite being associated with significant and severely toxic side effects due to the non-selectivity of antineoplastic drugs, which impact both malignant and normal tissues [7].

The modest effectiveness of existing treatments has led to significant investments in the development of new drugs. However, bringing a new drug to the market is a lengthy and costly process, with timelines averaging 11.4 to 13.5 years and costs ranging from USD 2 billion to USD 3 billion. About one in 5000–10,000 potential antineoplastic drugs receives FDA approval, with only 5% of oncology treatments progressing beyond Phase I clinical trials to gain final approval [8,9].

In this context, there has been a growing interest in “drug repurposing” in recent years. In this case, a repurposing or repositioning approach would be treating lung cancer with drugs that were originally developed and approved for other indications [10]. This strategy offers many advantages, but the main one is a faster progression through clinical trials at lower costs, facilitated by the use of drugs with already established pharmacokinetic and toxicity profiles. Statins, and simvastatin (SV) among them, are drugs commonly used to lower cholesterol levels and prevent cardiovascular diseases, as well as being widely studied for repositioning purposes, mainly due to their ability to inhibit oncogenic proteins from the Ras family [11,12]. However, their application as anticancer agents requires concentrations about 100 times higher than those used for cholesterol-lowering effects, thereby increasing the associated adverse effects such as liver dysfunction, myopathies, and rhabdomyolysis, among others [13].

Phytochemicals have also emerged as promising alternatives in cancer treatment, owing to their natural origin, low cost, and selective cytotoxicity toward tumor cells [14,15]. Monoterpenes found in essential oils have shown significant antitumor activity by inducing cell cycle arrest, autophagy, and apoptosis in in vitro and in vivo models [16,17,18,19]. Moreover, combining monoterpenes with conventional anticancer drugs has been reported to enhance their efficacy [20,21]. In addition to their effects on tumor cells, several monoterpenes have demonstrated positive anti-inflammatory and immunomodulatory effects, which are crucial aspects for the comprehensive treatment of cancer [22,23,24]. However, limitations associated with the low solubility and high volatility of terpenes frequently lead to a loss of bioavailability and effectiveness [25,26].

To overcome these challenges, nanotechnology has introduced advanced drug delivery systems such as solid lipid nanoparticles (SLNs) and nanostructured lipid carriers (NLCs) [27]. SLNs serve as colloidal drug carriers composed of a solid lipid matrix containing emulsifiers as stabilizers. However, SLNs may occasionally face challenges, including restricted capacity for loading, the potential for gelation, and the possibility of the molecular payload leaking as a result of lipid crystallization within the matrix over time [27,28].

On the other hand, owing to a biphasic structure that combines solid and liquid lipids, NLCs offer improved stability, higher drug loading capacity, encapsulation efficiency, and controlled release [27,28]. Various formulations of NLCs have been developed utilizing monoterpene oils as agents for dual nanostructuring and bioactivity [29,30]. As additional advantages, NLCs offer the benefit of encapsulating both water-insoluble and water-soluble compounds, are made from FDA-approved materials with low toxicity, and may be easily scaled up by implementing green technologies [28,31].

The current research focuses on developing bioactive NLCs containing phytocompounds as a novel biocompatible system designed to deliver and enhance the chemotherapeutic effects of the repurposed drug simvastatin against lung cancer cells.

## 2. Materials and Methods

### 2.1. Chemicals

The solid lipid Myristyl Myristate (MM, MW = 425 g/mol) and the liquid lipid Crodamol™ GTCC-LQ (CRD) were generously provided by Croda Argentina. Linalool (>95%, LN, MW = 154 g/mol, 5.588 M), limonene (97%, LM, MW = 136 g/mol, 6.18 M), 1,8-cineole (99%, CN, MW = 154 g/mol, 5.99 M), simvastatin (97%, SV, MW = 418 g/mol), tetrazolium dye MTT [3-(4,5-dimethylthiazol-2-yl)-2,5-diphenyltetrazolium bromide], rhodamine-123 (Rh-123), poloxamer 188 (Pluronic™ F68), 2,7-dichlorodihydrofluorescein diacetate (DCFH-DA), Propidium Iodide (PI), *N*-acetyl-l-cysteine (NAC), and 3,3-dioctadecyloxacarbocyanine perchlorate (DiOC18) were acquired from Sigma Chemical Co. (St. Louis, MO, USA). Dulbecco’s modified eagle medium (DMEM), Ham F12, fetal bovine serum (FBS), and penicillin-streptomycin (P/S) were provided by Gibco (Invitrogen Corporation, Waltham, MA, USA). Additional chemicals and solvents were sourced from Merck (Darmstadt, Germany), Carlo Erba (Milan, Italy), or equivalent suppliers.

### 2.2. Preparation of Lipid Nanoparticles

SV-loaded SLNs and NLCs were obtained through homogenization followed by the ultra-sonication method as described in previous reports [32]. In brief, MM was heated to 70 °C in a water bath, after which SV was added. For the preparation of NLCs, the liquid lipid (oil) was mixed into the molten lipid phase before incorporating SV. After 10 min, a temperature-controlled aqueous solution (20 mL) containing the surfactant poloxamer 188 was prepared and subsequently incorporated into the molten lipid phase. The resulting mixture was rapidly subjected to sonication (40% amplitude, 10 min) using an ultrasonic processor (130 W, Cole-Parmer, Vernon Hills, IL, USA) with a 6 mm titanium probe.

The resulting dispersion was allowed to cool at room temperature and then kept at 4 °C for further use. The different formulations and amounts of each ingredient are presented in Table 1.

The fluorescent-labeled nanoparticles were prepared as previously described plus the addition of 1.0 mg of DiOC18 (484/501 nm) [33,34]. DiOC18 is a green, fluorescent lipophilic carbocyanine dye commonly used as a lipophilic tracer. The fluorescent dye was added to the melted lipid, homogenized for 10 min, and the nanoparticles were subsequently prepared following standard methods. The probe demonstrated 100% encapsulation efficiency, with no detectable release under the experimental conditions tested.

For physicochemical analysis, the formulations were frozen at −80 °C and subsequently freeze-dried using a Rificor L-A-B4 lyophilizer (Buenos Aires, Argentina). The samples were then stored at room temperature in a dry desiccator to maintain stability [35].

### 2.3. Transmission Electron Microscopy (TEM)

The nanoparticle dispersion was diluted tenfold with ultrapure water, and a drop of the dispersion was placed onto a collodion-coated copper grid (400 mesh). Excess liquid was carefully removed using filter paper, and a drop of phosphotungstic acid was introduced to the nanoparticle dispersion to improve contrast. The sample was then analyzed in a JEOL 1200 EX II TEM microscope (JEOL, Peabody, MA, USA), as detailed earlier [34].

### 2.4. Particle Size, Zeta Potential, and Polydispersity Index

The mean diameter and size distribution (polydispersity index, PDI) of the NLC as well as the zeta potential (Z-pot) were determined in a Nano ZS Zetasizer instrument (Malvern Instruments Corp., Malvern, UK) at 25 °C, as previously described [36].

### 2.5. Cell Culture

The A549 human lung adenocarcinoma cells were obtained from the American Type Culture Collection (ATCC^®^ CCL-185™). Non-tumoral HaCaT keratinocyte cells (normal human adult skin) were a kind gift from Dr. Nahuel Ramella (School of Medicine, National University of La Plata). The cells were cultured in phenol-red DMEM supplemented with 10% FBS and antibiotics (1% penicillin/streptomycin) at 37 °C in a 5% CO_2_ atmosphere.

### 2.6. Cytotoxic Activity

The cytotoxic activity was evaluated by the MTT assay [23]. A549 (5 × 10^3^) and HaCaT (5 × 10^3^) cells were placed in a 96-well microplate and maintained at normal conditions for 24 h. Then, the cells were exposed to different experimental conditions and, after washing with phosphate buffer saline (PBS) twice, were exposed to 0.5 mg/mL MTT solution (in serum-free DMEM) over 3 h. Cell viability was determined by the MTT assay, as previously described [36].

### 2.7. Small- and Wide-Angle X-Ray Scattering (SAXS/WAXS) Studies: Encapsulation Efficiency

SAXS/WAXS measurements were performed in transmission mode using a XENOCS Xeuss 2.0 UHR HP200 instrument (XENOCS, Grenoble, France) equipped with a GeniX3D Cu ultra-low divergence microfocus X-ray source (average wavelength λ = 0.15419 nm) and two Dectris Pilatus3 R (DECTRIS, Baden-Dättwil, Switzerland) hybrid pixel photon-counting detectors: a 200K-A system for SAXS and a 100 K system for WAXS studies. The measured intensity, I, was plotted as a function of the modulus of the scattering vector, q, defined as q = 4π⁄λ sin (θ), where 2θ is the scattering angle. The sample-to-detector distance was 1200 mm for the SAXS detector, resulting in a q range of 0.085–3.50 nm^−1^. The WAXS detector was positioned at 158 mm with a 36° angle, providing a 2θ range of 15–45°. SAXS/WAXS patterns were taken for 30 min each. Data interpretation was divided into two aspects: Bragg peak identification and the study of the lamellar structure, following a procedure described in a previous report. The registered intensity, I(q), was recorded employing modulus of the scattering momentum transfer q, where q = 4π⁄λ sin (θ), 2θ is the scattering angle and λ = 0.15419 nm is the weighted average of X-ray wavelength of the Cu-Kα1,2 emission lines [37].

### 2.8. Encapsulation Efficiency (EE) and Drug Loading (DL)

The EE was determined by measuring the concentration of the unencapsulated drug, following the method described earlier [35]. After preparing the NLC, a 0.5 mL sample was placed into a centrifugal ultrafiltration unit (MWCO 3000, Microcon, Millipore, Burlington, MA, USA) and centrifuged at 10,000× *g* for 10 min at 25 °C using an Eppendorf MiniSpin microcentrifuge (Hamburg, Germany). Post-centrifugation, the concentration of free SV in the filtrate was measured by HPLC. The EE was then calculated using the following equation:

EE (%) = (Q_0_ − (C_r_ × V)/Q_0_) × 100

where Q_0_ is the initial amount of SV (in mg) in the formulation, C_r_ is the concentration of SV (mg/mL) in the filtrate, and V is the final volume of the formulation (mL).

Theoretical drug loading (DL, %) was calculated as follows:

DL (%) = (Mass of SV incorporated (mg)/Lipid mass (mg)) × 100

### 2.9. Analytical Quantification of SV and LN

SV and LN were quantified by an HPLC analytical method, employing a Dionex Ultimate 3000 UHPLC (Thermo Scientific, Sunnyvale, CA, USA) containing a dual gradient tertiary pump (DGP-3000) and a DAD-3000 diode array detector. Chromatographic separation used an ODS Hypersil RP18 column (100 × 4.6 mm, 5 μm, Thermo Scientific, Sunnyvale, CA, USA) as the stationary phase and a mixture of acetonitrile and 0.1% d-phosphoric acid (65:35) as the mobile phase. The chromatograph was isocratically operated at a flow rate of 1.0 mL/min, and detection was carried out at 238 nm for SV and 210 nm for LN.

### 2.10. Physical Stability

After nanoparticle synthesis, aliquots of the formulations were stored at 4 °C for 4 months, kept in the dark, and shielded from light. The physical stability of the nanoparticles was then assessed by measuring changes in average particle size, Z-pot, and PDI.

### 2.11. In Vitro Release Studies

The release behaviors of SV from SLN/SV, and LN and SV from NLC/LN/SV, were evaluated by equilibrium dialysis performed in an in-house device, developed by replacing the polycarbonate membrane of a 6-well plate (Costar Snapwell, Corning Inc., Corning, NY, USA) with a dialysis membrane (14 kDa molecular weight cut-off, Merck KGaA, Darmstadt, Germany). Briefly, 4 mL of release medium, consisting of isopropanol and water (30:70), was added to the acceptor compartment (lower chamber), while 0.2 mL of each sample was placed in the donor compartment (upper chamber), by duplicate. During the experiment, the plates were kept at 37 °C with shaking at 75 rpm, and 100 µL samples were taken at designated time intervals (from 0.25 to 72 h) and replaced with fresh medium at the same temperature. To reduce solvent evaporation during the test, the plate was sealed using a double mechanism: (1) sealing the plastic lid with tape all around, and (2) wrapping the system in plastic wrap. This strategy, along with fresh medium at the same temperature, allowed us to maintain sink conditions with low volume fluctuations. Finally, the SV and LN concentrations in the samples were quantified using HPLC, as previously described.

### 2.12. Hemolysis Studies

Heparinized venous blood from healthy donors was collected from volunteers. The blood was placed in a 12-well plate containing Ham F12 medium supplemented with 10% FBS with a final volume of 2 mL. Every culture was exposed to different concentrations of free LN, free SV, free LN + SV, or NLC/LN/SV and incubated for 1 and 3 h at 37 °C in a 5% CO_2_ atmosphere. After incubation, the mixture was centrifuged at 400× *g* for 5 min, and the pellet was removed. A 0.1 mL sample of the supernatant was then analyzed at 540 nm using a Multiskan™ GO spectrophotometer (Thermo Fisher Scientific, USA) to determine the extent of hemolysis. Erythrocytes exposed to 1.0% Triton X-100 served as the positive control for 100% hemolysis, while erythrocytes exposed to PBS were used as the negative control.

### 2.13. Static Incubation with FBS

The NLC/LN/SV dispersion (200 μL) was mixed with FBS (200 μL) to perform static incubation tests [38]. The mixture was incubated for 1 h at room temperature, then diluted 1:50 before DLS analysis. FBS and NLC/LN/SV solutions with identical final concentrations were used in the control experiments.

### 2.14. Interactions with Plasma Proteins Measured by Surface Plasmon Resonance

Measurements were conducted employing an MP- SPR Navi 210A system with incident light at 785 nm and a flow rate of 10 μL/min. In situ immobilization of proteins was obtained by a 20-min injection of 100 µg/mL fibrinogen solution in 10 mM sodium acetate (pH = 4.07), and 100 µg/mL human serum albumin (HSA) solution in 10 mM sodium acetate (pH = 4.4) over the gold SPR sensor in each three different individual experiments. Ten-fold dilutions of NLC/LN/SV in PBS were introduced into the device for 10 min, followed by the injection of PBS as the running buffer. Control experiments corresponded to the injection of NLC/LN/SV dilutions over the sensors where no protein was immobilized.

### 2.15. Cellular Uptake

The cellular uptake of the nanoparticles (NPs) was carried out using the fluorescent probe DiOC18 (λ = 484/501 nm), as described earlier [32]. Briefly, A549 cells (5 × 10^3^) seeded in 96-well plates were exposed to DMEM supplemented with FBS containing NLC/LN/SV/DIOC18 (5 and 10 μM SV) for 1, 3, and 6 h. Then, the cells were rinsed with PBS three times, and fluorescence was measured in a Beckman Coulter DTX 880 microplate reader (Brea, CA, USA).

### 2.16. Determination of Reactive Oxygen Species

Intracellular reactive oxygen species (ROS) production was measured using DCFH-DA (Sigma Aldrich, St. Louis, MO, USA), a peroxide-sensitive fluorescent probe [18,19]. In brief, A549 cells were plated in 96-well plates (5 × 10^3^) and exposed for 24 h to ethanol 0.2% (Control), free SV (10 μM), and NLC/LN/SV (5 and 10 μM of SV). After that, cells were stained with 10 μM DCFH-DA dissolved in serum-free DMEM for 30 min at 37 °C in darkness. Following three PBS washes, the fluorescence was measured in a microplate reader (485 ex/535 em).

To determine the influence of ROS on A549 cell viability, the cells were pre-treated with 5 mM NAC for 2 h before co-treatment with NLC/LN/SV for 24 h. Cell survival was determined by MTT.

### 2.17. Evaluation of Mitochondrial Membrane Potential

Mitochondrial membrane potential (MMP) was evaluated using the fluorescent probe Rh-123, a cationic dye that selectively accumulates within mitochondria based on MMP [39]. A549 cells (5 × 10^3^) were plated in 96-well plates and treated with ethanol 0.2% (Control), free SV (10 μM), and NLC/LN/SV (5 and 10 μM of SV) for 24 h. After three washes with PBS, cells were incubated with 10 μM Rh-123 in serum-free DMEM (30 min, 37 °C, darkness). Subsequently, cells were washed three more times with PBS, and fluorescence intensity was measured in a microplate reader (485-ex/535-em, Beckman Coulter DTX 880, Brea, CA, USA).

### 2.18. Cell Cycle Analysis

A549 cells (1.5 × 10^5^) were plated in 6-well plates and grown for 24 h. Following treatments, the cells were trypsinized, centrifuged for 5 min at 500× *g*, resuspended in PBS, and fixed overnight in 70% ice-cold ethanol. After washing twice with PBS, cells were exposed to Ribonuclease A (0.1 mg/mL, Sigma Aldrich) for 30 min at 37 °C. The nuclei of the cells were stained with PI (0.025 mg/mL) in darkness for 30 min, followed by analysis using flow cytometry (BD Accury C6 Plus, BD Biosciences, Franklin Lakes, NJ, USA). The data were then processed using FlowJo version X 10.0.7r2 (Tree Star, Ashland, OR, USA).

### 2.19. Cell Death

A549 cells (3 × 10^4^) were placed in a 24-well plate and allowed to adhere for 24 h. Following the treatments, the supernatant was collected, and the attached cells were harvested by trypsinization, centrifuged at 500× *g* for 5 min, resuspended in 0.1 mL of complete DMEM, and combined with the supernatant fraction. The resulting cell suspensions were mixed in a 1:1 (*v*/*v*) ratio with a 0.4% (*w*/*v*) trypan blue solution. Cell death was assessed by counting non-stained (live) and blue-stained (dead) cells using a Neubauer hemocytometer, as previously described [19].

### 2.20. Wound Healing Assay

A549 cells (7.5 × 10^4^) were planted in 24-well plates and allowed to adhere for 24 h. A wound was created using a sterile yellow tip, and detached cells were removed by washing with serum-free DMEM. Thereafter, cells were treated with ethanol 0.2% (control), free SV (10 μM), or NLC/LN/SV (5 and 10 μM SV) in serum-free DMEM for 48 h. Cell migration was assessed at 0 and 48 h post wounding employing an Olympus LX71 Inverted Microscope (Tokyo, Japan). The scratched area was quantified using ImageJ 1.53k software (National Institute of Health, Bethesda, MD, USA) [36].

### 2.21. Statistical Analysis

Results are expressed as the means ± standard deviation (SD). When appropriate, analysis of variance (ANOVA) followed by the Tukey–Kramer multiple comparisons or the unpaired *t*-test were employed. A significance level of 0.05 was used. Non-linear regression curves generated with SigmaPlot software (version 14.0; Systat Software, Inc., Point Richmond, Richmond, CA, USA) were used to calculate IC_50_ values for cell viability.

## 3. Results and Discussion

### 3.1. Synthesis of Lipid Nanoparticles

In this study, two types of lipid nanoparticles were produced: solid lipid nanoparticles (SLNs) and nanostructured lipid carriers (NLCs), distinguished by the absence or the incorporation of liquid lipids (oils) into the lipid matrix, respectively. In addition, different formulations were obtained considering the incorporation of SV in combination with the oils (Table 1).

The composition of the SLN/NLC formulations was based on previous work conducted in our laboratories [33,35,36,40,41,42]. Specifically, in prior research, our group extensively analyzed the impact of key parameters—including initial lipid content, surfactant concentration, and the addition of oils—on NLC formation [43]. The influence of oil/hydrophobic molecules incorporation was also systematically investigated in several studies [33,35,36,42].

**Figure 1:**
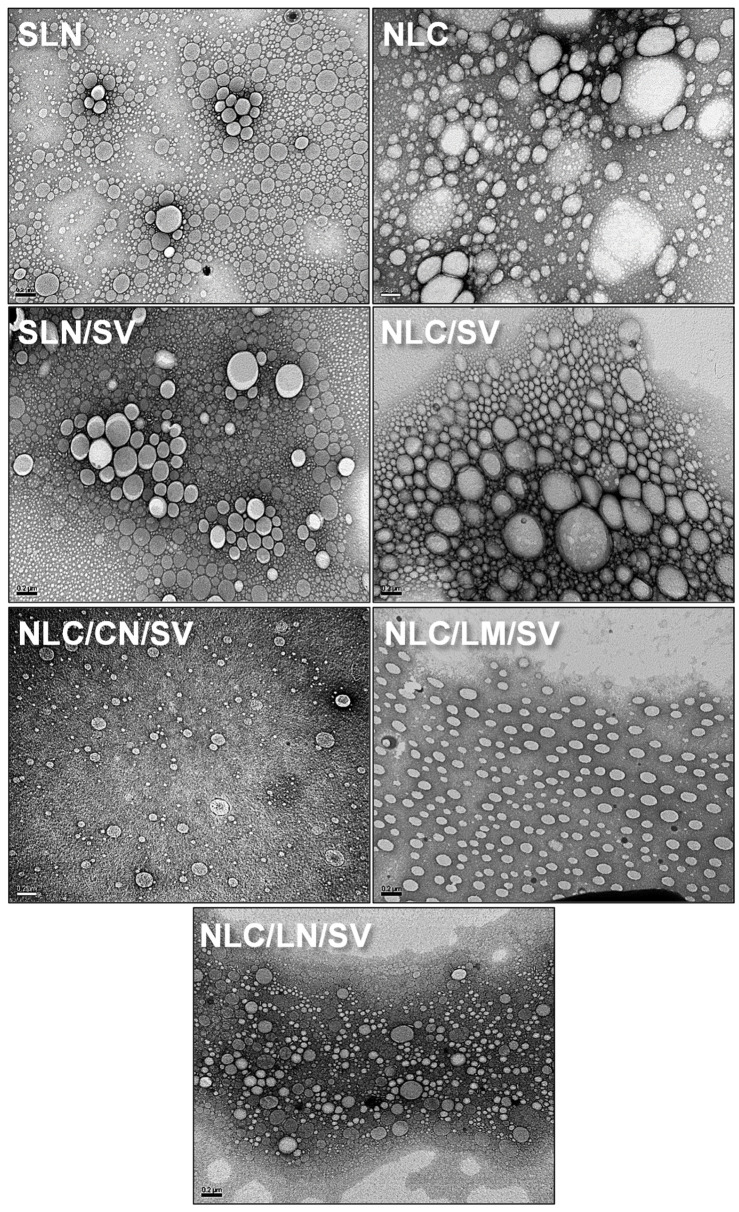
TEM images of SLN, NLC, SLN/SV, NLC/SV, NLC/CN/SV, NLC/LM/SV, and NLC/LN/SV. Scale bar: 200 nm.

For NLC development, we employed either a commercial, non-bioactive CRD liquid lipid (standard NLC) or bioactive monoterpenes (LN, LM, and CN) as oils. MM was selected as the solid lipid matrix for nanoparticle synthesis considering its compatibility with both the lipophilic nature of SV and the monoterpenes (LN, LM, and CN), as was previously explored with similar cargo molecules [32,33,35,36,41,44]. As evidenced by transmission electron microscopy (TEM) images, all the formulations (i.e., SLN, NLC, SLN/SV, NLC/SV, NLC/LN/SV, NLC/LM/SV, and NLC/CN/SV) consisted of homogeneous, spherical nanoparticles (Figure 1). As previously demonstrated by our group, MM has proven to be effective in producing stable colloidal nanoparticles [33,34].

The results from the TEM images were further corroborated by dynamic light scattering (DLS) analysis (Table 2), which provides a more appropriate and less invasive analysis method than TEM for NPs suspended in aqueous environments [36]. Mean particle diameters range from 95 to 145 nm, with PDI between 0.17 and 0.25, and Z-pot in the range of −4.5 to −16.3 mV. Particularly, the incorporation of LN produced a significant reduction in the mean size of the nanoparticles compared with SLN/SV or NLC/SV (94.2 nm vs. 130.9 nm and 142.4 nm, respectively, *p* < 0.001). This size reduction may be attributed to the physicochemical properties of LN relative to other oils, including its chemical functional groups and interactions with MM. This effect was previously reported in a study from our laboratory, where nanoparticles were physiochemically characterized by FTIR, XRD, DSC, and TGA [33]. In that study, among the formulations tested, SLN-MM exhibited the smallest particle diameters (118 nm for empty SLN and 92 nm for LN-loaded SLN). These results suggested that loading LN into the lipid matrix induced changes in the polymorphic state of the lipid, transitioning from crystalline to amorphous. Furthermore, a reduced crystallinity of the nanoparticles was observed and evidenced by their lower energy requirements for melting, indicating a less-ordered structure compared to crystalline forms after DSC and TGA analysis. FTIR analysis supported these findings, revealing minor peak shifts (1–3 cm^−1^) following LN incorporation, indicative of weak interactions between LN and the MM lipid matrix, possibly involving hydrogen bonds and/or hydrophobic interactions. Also, XRD analysis showed that the SLN-MM exhibited sharp peaks at 2θ angles of 19.1°, 20.7°, 21.6°, 23.3°, and 23.9°, confirming the lipid’s crystalline nature. However, the absence of the peak at 20.7° after LN incorporation suggested alterations to the crystal structure, resulting in a less-ordered matrix [33].

Other works also determined that the impact of LN on particle size is critical for the stability, bioavailability, and application potential of these formulations. Smaller particle sizes are generally associated with enhanced stability against gravitational separation and aggregation, as well as improved penetration and efficacy of active compounds [45].

Since all formulations exhibited a particle size below 200 nm, which facilitates passive accumulation in the tumor microenvironment via the enhanced permeation and retention (EPR) effect [46], alongside a PDI less than 0.3 indicating excellent compatibility for biomedical applications [47], the cytotoxic activity of these formulations was subsequently evaluated in A549 lung cancer cells.

### 3.2. Screening of Best Formulations Based on Cytotoxic Activity of Free and Encapsulated SV into Lipid Nanoparticles

We have previously reported that SV presents an IC_50_ of 20–25 μM and that non-cytotoxic concentrations of SV (10 μM) combined with monoterpenes (also at suboptimal levels) synergistically inhibited A549 cell growth [19,48]. To investigate whether encapsulation of SV into different bioactive NLC (containing LN, LM, or CN) may increase its cytotoxicity compared to free or standard SLN and NLC formulations, A549 cells were exposed to free SV, SLN, NLC, SLN/SV, NLC/SV, NLC/LN/SV, NLC/LM/SV, and NLC/CN/SV (10 μM SV in all cases, and equivalent amounts of lipid content for NPs without SV).

**Figure 2:**
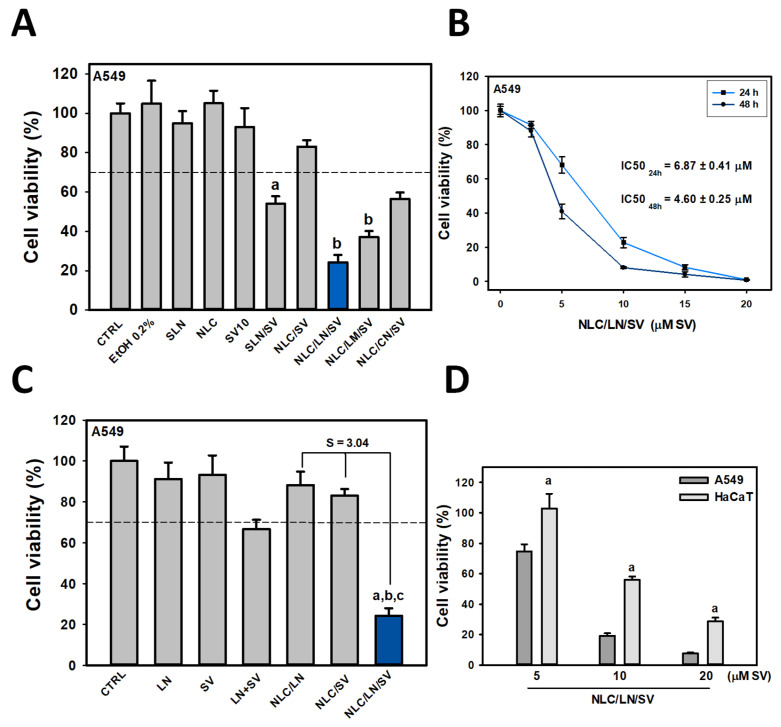
Cytotoxicity of the different formulations on A549 lung cancer cells. (**A**) A549 cells were exposed to 10 µM SV (free or encapsulated), EtOH 0.2% (vehicle for free SV and monoterpenes), and equivalent amounts of empty NPs (SLN and NLC) for 24 h. Results are expressed as the mean ± SD (*n* = 8), (a) *p* < 0.001 vs. non-treated cells (CTRL), (b) *p* < 0.001 vs. SLN/SV. The blue bar (NLC/LN/SV) represents the most cytotoxic formulation. The dotted line represents 70% viability; below which the formulation may be considered cytotoxic. (**B**) A549 cells were exposed to increasing concentrations of NLC/LN/SV for 24 and 48 h. IC_50_ values were calculated as described in Section 2.21. (**C**) Cells were exposed to 900 µM LN or 10 µM SV (free or encapsulated), and their combination, LN + SV (free), or NLC/LN/SV (encapsulated) for 24 h. Synergistic effect (S) was calculated. S > 1 indicated synergism with respect to individual encapsulated LN (NLC/LN) and SV (SLN/SV). (a) *p* < 0.001 vs. free SV, (b) *p* < 0.001 vs. LN + SV; (c) *p* < 0.001 vs. NLC/SV. The blue bar (NLC/LN/SV) represents the most cytotoxic formulation. The dotted line represents 70% viability; below which the formulation may be considered cytotoxic. (**D**) Normal HaCaT and A549 lung cancer cells were exposed to increasing concentrations of NLC/LN/SV (5, 10, and 20 μM SV) for 24 h. (a) *p* <0.001 vs. HaCaT cells.

As expected, free SV and biocompatible empty SLN and NLC formulations showed no cytotoxic effects (Figure 2A). However, encapsulating SV into standard lipid NPs significantly increased its cytotoxicity, with a more pronounced effect observed in SLN/SV (54.0% cell viability, *p* < 0.001). This observation may be associated with a faster and less controlled release of SV from SLN compared to NLC [27,28], which may become more evident during relatively short incubation periods. Finally, bioactive NLCs containing monoterpenes were able to exacerbate SV cytotoxicity compared to NLC/SV, decreasing cell viability from 83.0% (NLC/SV) to 24.2% (NLC/LN/SV), 37.1% (NLC/LM/SV), and 56.5% (NLC/CN/SV), respectively (Figure 2A, *p* < 0.001), which highlight the relevance of employing bioactive monoterpenes as nanostructuring oils. A combination of various factors, such as synergism, differential release of monoterpenes and/or SV, and even particle size, could be involved in the enhanced cytotoxicity of NLC/LN/SV compared to NLC/LM/SV and NLC/CN/SV. Indeed, nanoparticle uptake in cancer cells increases as particle size decreases, regardless of the content or functional groups on the particle surface [49,50]. Furthermore, NLC/LN/SV exhibits an optimal particle size for cancer therapy. Nanoparticles smaller than 10 nm can pass through normal blood vessel walls and undergo renal excretion, while those larger than 100 nm tend to be cleared by the liver and spleen. Then, nanoparticles 20–100 nm in size offer several advantages: extend drug circulation times to improve bioavailability and reduce required dosages, and enhance targeted tumor delivery, minimizing the risk of nonspecific organ toxicity [51].

It is important to note that monoterpenes encapsulated alone at the same concentrations (NLC/CN, NLC/LM, and NLC/LN) were not cytotoxic for A549 cells (Figure S1). Based on these results, as well as those obtained by TEM and DLS, considering the ideal particle sizes for cancer therapy [51], the NLC/LN/SV formulation was selected as the most promising system for further studies.

### 3.3. Biological Evaluation and Physicochemical Characterization of NLC/LN/SV

NLC/LN/SV was shown to inhibit A549 cell viability in a dose- and time-dependent manner, and IC_50_ values after 24 and 48 h exposition were calculated (Figure 2B). NLC/LN/SV was shown to potentiate 2.2-fold the cytotoxic effect of free LN + SV at equivalent concentrations, whereas a strong synergistic effect (S = 3.04) was observed for NLC/LN/SV in comparison to the sum of the LN and SV individually encapsulated (SLN/LN and NLC/SV) (Figure 2C). A main goal in cancer treatment is to target tumor cells while minimizing or preventing toxic side effects on healthy tissues. For that purpose, NLC/LN/SV cytotoxicity was evaluated in both A549 lung cancer and normal HaCaT cells [52]. As shown in Figure 2D, NLC/LN/SV significantly reduced A549 cell viability by 25%, 79%, and 93% after 5, 10, and 20 μM SV exposure, respectively, compared to reductions of 0%, 44%, and 71% in HaCaT cells (*p* < 0.001). These results suggest that NLC/LN/SV exhibits selectivity toward cancer cells.

Several factors may contribute to the selective cytotoxicity of NLC/LN/SV. Ras GTPase is a critical therapeutic target in NSCLC, as RAS gene activating mutations are common oncogenic alterations in NSCLC, including A549 cells [53]. Diverse treatment options targeting RAS mutations in NSCLC have been developed with acceptable toxicity toward normal tissues [54]. We have previously shown that LN combined with SV strongly reduced Ras anchorage in the cell membrane of cancer cells [55], essential for its oncogenic activity. In addition, cancer cells exhibit elevated ROS levels compared to normal cells, driven by increased mitochondrial metabolic activity, electron transport chain disruptions, and oncogenic signaling [56,57]. These elevated ROS levels contribute to tumorigenesis, angiogenesis, metastasis, and therapy resistance. While moderate ROS levels support tumor progression, excessive ROS induces cell death, making ROS induction a key target for NSCLC anticancer agents [56,57], including SV and LN [19,58]. These mechanisms likely contribute to the enhanced cytotoxicity of NLC/LN/SV against A549 cancer cells, although further investigation is needed to completely understand this effect.

**Figure 3:**
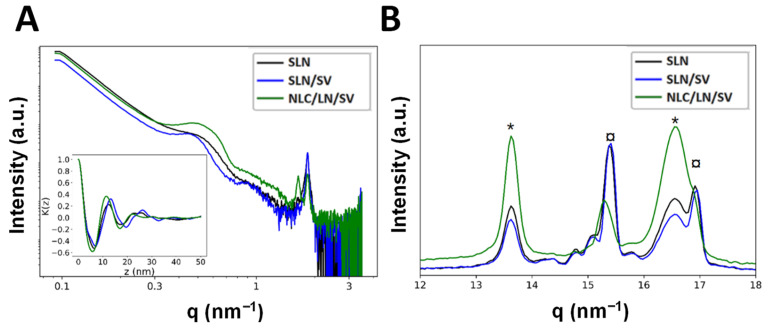
(**A**) SAXS patterns of three different formulations (SLN, SLN/SV, and NLC/LN/SV). The inset shows the 1D correlation transformation related to the stacking periodicity of the copolymer assembly. (**B**) WAXS patterns of the same three formulations, where (*) indicates the copolymer diffraction contribution while (¤) indicates the lipid phase diffraction peaks.

To reveal the role of SV and LN in the assembly of the lipid matrix (primarily containing MM), (SAXS/WAXS) analysis was carried out for SLN, SLN/SV, and NLC/LN/SV formulations. SAXS (Figure 3A) and WAXS (Figure 3B) patterns revealed the presence of a Bragg peak corresponding to the β’ family type polymorph of myristyl myristate (MM) in the NP core, along with contributions from the copolymer. Consistent with previous studies, MM undergoes polymorphic transitions when interacting with lipophilic molecules such as monoterpenes [32]. However, in this study, the inclusion of SV did not induce any noticeable changes in the MM polymorphs. A change in the supramolecular structure of the copolymer was observed instead. The lamellar-like structure of the copolymer remained like that of pure Poloxamer 188 within the solid lipid nanoparticles (SLNs), albeit with increased PDI (Table 3), which may be attributed to sample processing conditions. SV led to an increase in both lamellar thickness and crystallinity, suggesting strong interactions between the drug and the surface of the SLN, rather than the core. In contrast, the addition of LN exhibited a distinct effect, decreasing the long period and crystalline thickness while increasing polydispersity. Considering that the bioavailability of drugs is influenced by their location within the nanoparticle architecture, variations in this parameter could significantly impact the release profile of the encapsulated molecules [59]. These findings suggest that LN significantly impacts both the core and shell of the lipid nanoparticles, potentially influencing the drug bioavailability.

**Figure 4:**
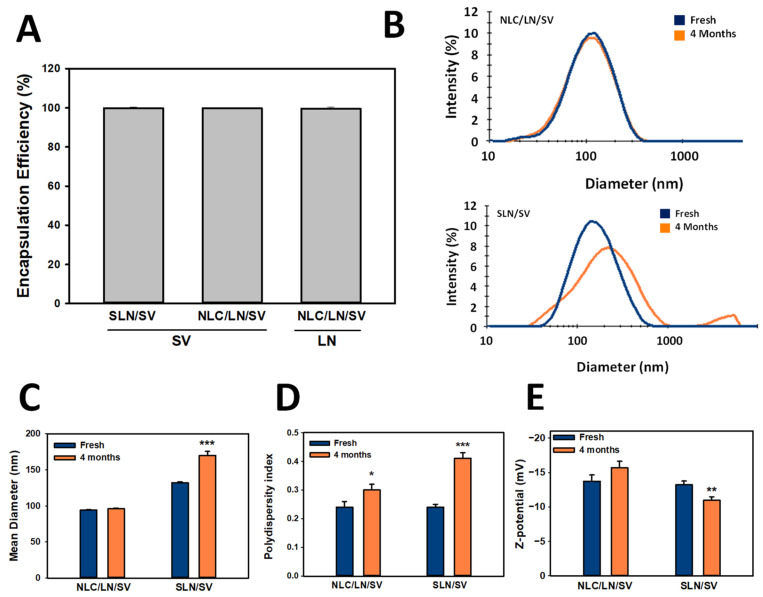
Encapsulation efficiency (EE), release profile, and stability of nanoparticles (**B**–**E**). (**A**) Encapsulation efficiency of SV and LN into SLN/SV and/or NLC/LN/SV. Stability of SLN/SV and NLC/LN/SV over time in terms of size distribution (**B**), mean size (**C**), PDI (**D**), and Z-pot (**E**). The results express the mean ± SD (*n* = 3). (*) *p* < 0.05; (**) *p* < 0.01; (***) *p* < 0.001.

The EE of SV (in SLN/SV and NLC/LN/SV) and LN (in NLC/LN/SV) was evaluated as previously reported by our group [32,36], and LN and SV detected by HPLC, as described previously. As observed in Figure 4A, both SV and LN were efficiently encapsulated (>95%) in the formulations, in concordance with the higher EE obtained for LN and other lipophilic monoterpenes in similar lipid nanoparticles [32,33,34] and reported for SV elsewhere [60]. Considering that the SV EE for SLN/SV and NLC/LN/SV formulations was close to 100%, the corresponding DL was calculated as 3.15%.

The stability of a formulation over time is a crucial factor for ensuring its reproducibility and potential application. Here, stability was monitored over 4 months in terms of particle size, PDI, and Z-potential of the formulations (SLN/SV and NLC/LN/SV) stored at 4 °C (Figure 4B–E). Of note, NLC/LN/SV were highly stable for up to 4 months, in contrast to SLN/SV, characterized by the appearance of a population with sizes higher than 1000 nm (aggregates, Figure 4B), the increase in both the mean diameter, from 130 nm to 170 nm, and PDI, from 0.24 to 0.41, respectively (Figure 4C,D). The greater stability of NLC/LN/SV may be associated with a surfactant-like behavior of LN, as we previously reported for similar monoterpenes [32], which may contribute to better stability of the formulation [61].

**Figure 5:**
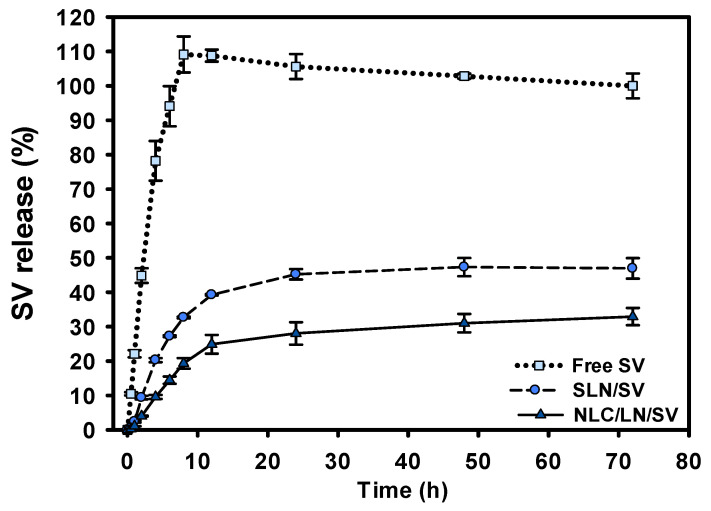
Release profile of SV from SLN/SV and NLC/LN/SV. The release behavior of SV was evaluated by equilibrium dialysis (14 kDa molecular weight cut-off) in a release medium consisting of isopropanol and water (30:70) at 37 °C and 75 rpm. The SV was quantified by the HPLC analytical method (238 nm). The graph represents the mean ± SD (*n* = 2) of each time point.

The release behavior of SV from the SLN/SV and NLC/LN/SV formulations was then evaluated (Figure 5). Due to the low aqueous solubility of SV, its release profile needed to be assessed in aqueous media containing either surfactants or organic solvents [62]. Surfactants were excluded in this study because they interfered with the passage of the free drug through the dialysis membrane. Instead, a 30% isopropanol solution in water was used. The 30% isopropanol concentration was chosen, as it was the minimum proportion, based on the media listed in the USP dissolution methods database [63], that ensured sink conditions during the test. Sink conditions, where the drug concentration in the dissolution media does not exceed 20% of its solubility, are necessary for obtaining biorelevant results [64]. In these conditions, free SV was completely released after 8 h. The initial burst release of SV, in the first 12 h, from both formulations may be attributed to the presence of drug molecules positioned on the surface of the nanoparticles. This effect was more pronounced in SLN/SV, with 39.2% of SV released in 12 h, compared to 24.9% of SV released from NLC/LN/SV. Then, a sustained and controlled release of SV was observed, reaching 46.9% for SLN/SV and 32.9% for NLC/LN/SV after 72 h. These findings align with those observed in SAXS/WAXS analysis and are consistent with those expected for the second-generation NLC, in terms of preventing or minimizing the possibility of systemic toxicity due to the leakage of the SV in the bloodstream before the nanoparticles have reached the tumor cells [27].

Additionally, we monitored the LN release from NLC, which may play an important role in the anticancer activity. Although not presented in Figure 5, LN was entirely released from the nanoparticles within the first 8 h. The difference in the release rate between LN and SV could be explained by the difference between the partition coefficient (logP) of these drugs (2.67 and 4.46 for LN and SV, respectively) [65,66]. The higher lipophilicity of SV may allow it to better accumulate in the lipid matrix, decreasing the release rate in comparison to LN [67]. Nevertheless, the rapid release of LN could be an advantage of these nanoparticles, as it could give an initial antitumor effect due to this terpene and then slowly release SV at the tumor site. Also, the effective cytotoxic impact of the nanoparticles on cancer cells is expected to result from the combination of an optimal release profile and efficient cellular uptake [50,68]. Despite the influence of pH on drug release, NLC cellular uptake remains an important and complementary parameter to evaluate, as it represents the primary mechanism of internalization for lipid nanoparticles with similar characteristics, predominantly via the endocytic pathway [50,68,69].

### 3.4. NLC/LN/SV Biocompatibility

**Figure 6:**
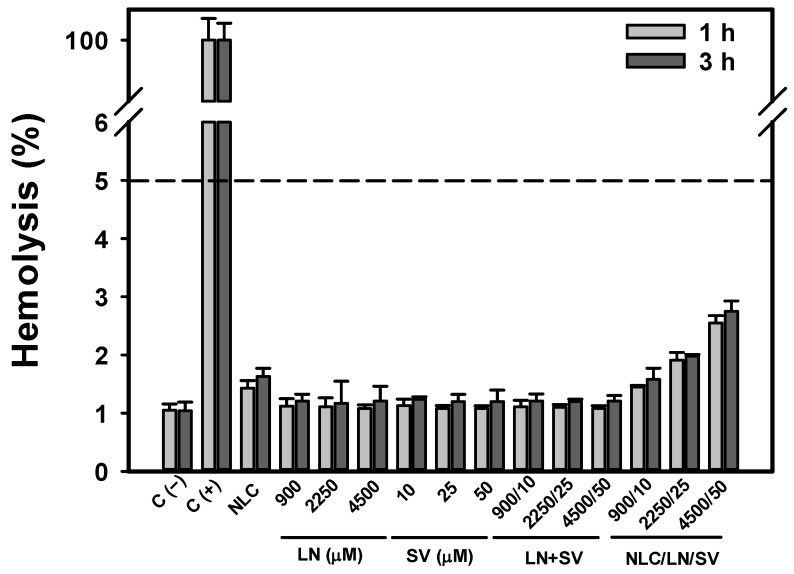
Percentage of hemolysis produced by empty NLC, free LN, SV, LN + SV, and NLC/LN/SV. Hemolysis of SV (10, 25, and 50 µM), LN (900, 2250, and 4500 µM), and the respective combinations (free or encapsulated), and equivalent quantities of empty SLN was measured after 1 and 3 h exposition. The results are expressed as the mean ± SD (*n* = 3). C (−): non-treated cells; C (+): erythrocytes lysed with 1.0% Triton X-100; SLN: empty nanoparticles. The dotted line (5% hemolysis) indicates the maximum accepted for human applications (ISO/TR 7406 standard [71]).

The elimination of tumor cells while avoiding or reducing detrimental side effects on healthy tissues is a crucial aspect of cancer treatment. Therefore, as a first approach, the hemocompatibility of NLC/LN/SV (Figure 6) was assessed. Considering intravenous administration as a potential route for NLC/LN/SV delivery, the interaction of the NPs with red blood cells becomes a potential issue of concern [70]. Erythrocytes were exposed to concentrations up to five-fold those stipulated as optimal against A549 cells for NLC/LN/SV (50 μM SV), empty NLC, as well as the free drugs alone (LN or SV) or in combination (LN + SV). Blood sample analysis showed negligible hemolysis, with levels below 3% in all cases, which is considered safe for human applications according to the ISO/TR 7406 standard [71]. Notably, although not biologically significant, a dose-dependent effect on erythrocytes was observed for NLC/LN/SV, reaching 2.7% hemolysis at the highest tested concentration. We hypothesized that the excess of both the surfactant P188 and LN may lead to the formation of small micelles [33], which are capable of subtly destabilizing the membrane of erythrocytes.

These findings, along with the results observed in normal HaCaT cells (Figure 2D), indicate that NLC/LN/SV can serve as a safe and biocompatible drug delivery system, able to enhance the anticancer activity of SV.

### 3.5. Formation of Protein Corona and Interaction of NLC/LN/SV with Plasma Proteins

**Figure 7:**
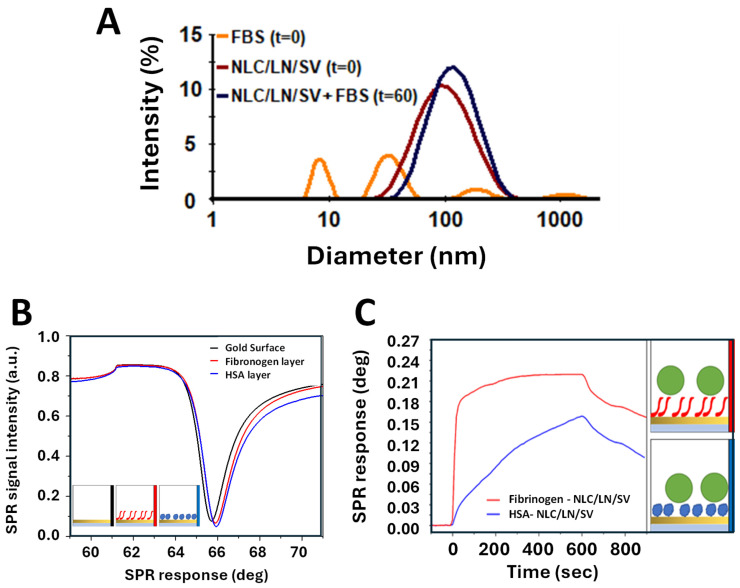
Interaction of NLC/LN/SV with plasma proteins. Creation of protein corona and interaction with specific plasma proteins. (**A**) DLS distributions of FBS (yellow) and NLC/LN/SV (red) at t = 0 min, and NLC/LN/SV–protein corona (blue) at t = 60 min. (**B**) Measured full SPR curves before and after protein immobilization. (**C**) SPR sensorgrams showing NLC/LN/SV binding to immobilized plasma proteins. The measurements were performed in the Angular Scan mode of MP-SPR.

The dynamic interaction between NPs and surrounding proteins in a biological context can lead to the formation of a protein corona (PC), a collection of proteins attached to the NP’s surface. In turn, PC determines the pharmacokinetics, toxicity, and stability of the NPs and may influence their targeting properties and their plasmatic clearance [72]. Therefore, we first evaluated whether NLC/LN/SV exposed to FBS under static conditions modified the mean hydrodynamic diameter (MHD) of the particles. Before incubation, the NLC/LN/SV formulation presented a unimodal and centered DLS distribution at MHD = 94 ± 1 nm, while the FBS solution showed a trimodal DLS distribution, characterized by peaks centered at 8 nm, 32 nm, and 220 nm attributable to representative serum protein sizes (Figure 7A). After incubation with FBS for 60 min, a single peak centered at MHD ≈ 101 nm ± 1 was observed (Figure 7A). The observed shift of ~7 nm in the MHD of NLC/LN/SV is compatible with a single layer of low- and medium-MHD serum proteins, whose peaks were not detected after 60 min FBS incubation [73]. Moreover, these findings suggest that no clustering processes occur in the formulation in the presence of serum, unlike lipid NPs of similar size that exhibited a ~200 nm shift after FBS incubation under identical conditions [38]. Finally, it is important to note that the mean MHD of ~100 nm obtained for NLC/LN/SV after FBS incubation fits with the optimal conditions required for passive targeting of NPs to solid tumors through the EPR effect [46].

As a complement to the preliminary evaluation of the interaction of NLC/LN/SV with plasma proteins, HSA, and fibrinogen were immobilized by physical adsorption onto a gold sensor surface (Figure 7B). NLC/LN/SV suspensions were injected across the functionalized surfaces to investigate the biomolecular interactions. Sensorgrams, i.e., plots of SPR response against time, showing the progress of the interaction, are shown in Figure 7C. Typical one-to-one exponential sensorgrams were observed [74], but the kinetics were different. For fibrinogen–NLC/LN/SV interaction, the association phase (increasing part of the curve) has a fast association rate, and the dissociation region (decreasing part of the curve) exhibits a relatively slow dissociation rate, whereas for HSA–NLC/LN/SV interaction, the sensorgram exhibits moderate association and dissociation rates. The observed differences in the kinetics of the 1:1 binding of fibrinogen–NLC/LN/SV and HSA–NLC/LN/SV imply a stronger interaction of the NPs with fibrinogen. These results raise potential concern regarding the stability of the formulation, as they may preliminarily suggest a tendency of NLC/LN/SV to undergo rapid clearance from serum, similarly to what was reported for polycatecholamine-coated nanoparticles [75]. Nevertheless, the actual efficiency as opsonins for phagocytic cells must be analyzed in future in vivo experiments where the NPs are exposed to the complete set of serum proteins, as the top three most abundant corona proteins in lipid NPs are apolipoproteins [76]. These results might also provide insights into the growing use of nanoparticles interacting with coagulation proteins in hemostatic applications [77].

### 3.6. Cellular Uptake of NLC/LN/SV

**Figure 8:**
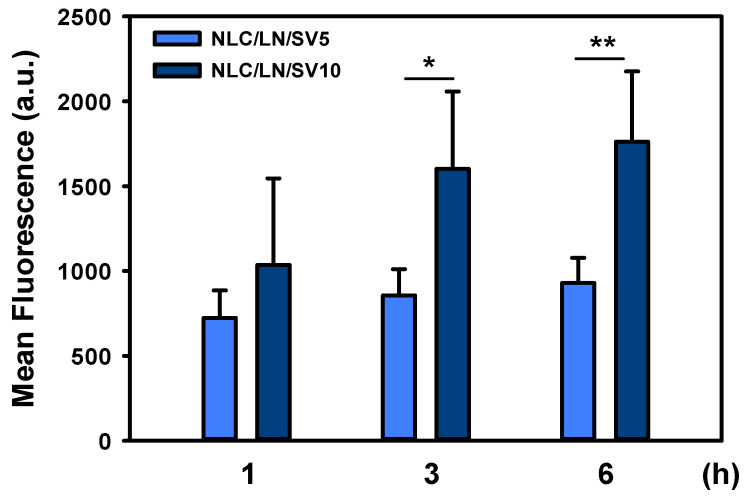
Cellular uptake of NLC/LN/SV in A549 cells. Cells were incubated with DiOC18-loaded NLC/LN/SV (5 or 10 μM SV) for 1, 3, or 6 h. Fluorescence intensity was measured spectrophotometrically. The results are the mean ± SD (*n* = 4). (*) *p* < 0.05; (**) *p* < 0.01.

The cellular uptake profile of NPs is an important tool for predicting a nanomedicines’ delivery efficiency and bioavailability and is characterized by the specific or non-specific adhesion of the particles to the cell surface, followed by their internalization through mechanisms such as endocytosis or pinocytosis [78]. The fluorescent green dye DiOC18 was used because it is irreversibly loaded and does not diffuse out from the NLC, which guarantees that the fluorescence indicates the localization of NPs [79]. NLC/LN/SV cellular uptake was evaluated in experimental conditions where cell viability was not significantly affected (up to 6 h, 10 μM SV). It was observed that A549 cells were able to incorporate the NLC (Figure 8). Time- and dose-dependent uptake of NLC/LN/SV was observed. Indeed, the dose-dependent uptake was more evident at 3 and 6 h incubation, where NLC/LN/SV/DiOC18 nearly doubled the green fluorescence at SV 10 μM compared to SV 5 μM (*p* < 0.05 at 3 h, and *p* < 0.01 at 6 h, respectively). This is an important finding, as it shows that NLC/LN/SV can enter into the cells and transport both SV and LN intracellularly, a characteristic that we have previously described for similar formulations [32,33].

### 3.7. Anticancer Mechanisms of NLC/LN/SV in A549 Cells

#### 3.7.1. Effects of NLC/LN/SV in ROS Production

**Figure 9:**
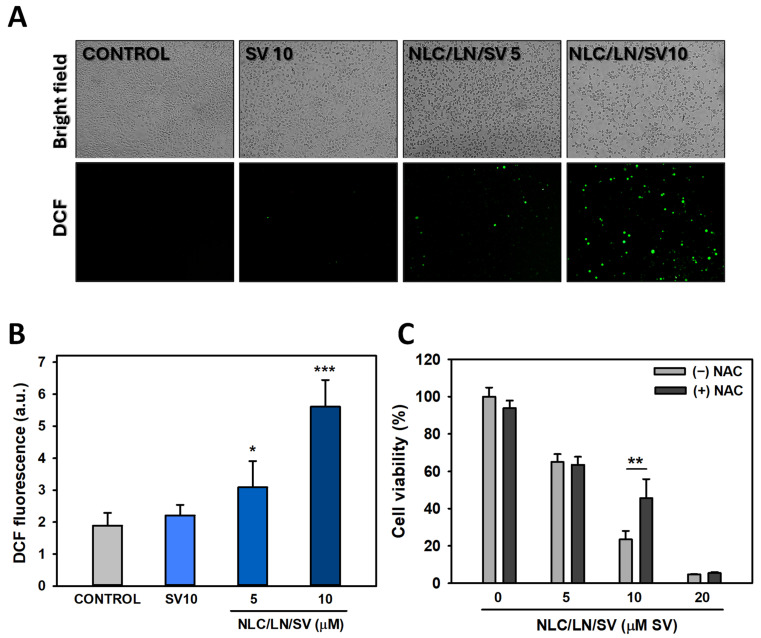
Effects of NLC/LN/SV on intracellular reactive oxygen species (ROS) in A549 cells. (**A**) Cells were exposed to ethanol 0.2% (control), SV 10 µM, or NLC/LN/SV at 5 and 10 µM SV for 24 h (100×). (**B**) ROS generation was evaluated under an inverted fluorescence microscope, and fluorescence intensity was quantified employing ImageJ 1.53 k (a.u. = arbitrary units). (**C**) Effect of the antioxidant compound NAC on A549 cell viability. Cells were treated or not with NAC 5 mM for 2 h before the addition of ethanol 0.2% (control) or NLC/LN/SV (5, 10, and 20 µM) for 24 h. Cell viability was measured by the MTT assay. Data are presented as the mean ± SD (*n* = 6). (*) *p* < 0.05, (**) *p* < 0.01, (***) *p* < 0.001.

As the next step, the induction of ROS production by NLC/LN/SV was studied by fluorescence microscopy (Figure 9A). ROS are essential for several signaling pathways that determine the fate of both healthy and cancer cells [80]. Intracellular ROS are produced at physiological levels in normal cells, whereas cancer cells typically exhibit higher ROS levels, contributing to tumorigenesis, angiogenesis, metastasis, and tumor resistance processes. ROS levels elevated to toxic thresholds trigger ROS-related cell death mechanisms. A usual strategy followed by numerous chemotherapeutic agents involves inducing ROS production to destroy cancer cells. Whereas modest ROS promotion may lead to cytostatic effects, excessive ROS promotes cytotoxic cell death [81]. Here, we found that NLC/LN/SV significantly induced ROS generation at 5 μM SV (~1.6-fold, *p* < 0.05) and 10 μM SV (~3.0-fold, *p* < 0.001) compared to the control without SV (Figure 9B). Moreover, free SV 10 μM did not affect ROS generation in the same conditions. These results may be the sum of both encapsulation of SV and pro-oxidant additive/synergistic activity when combined with LN, which can induce ROS production in A549 cells, as we previously reported [19]. It has been reported by our group and others that LN induces mitochondrial ROS production [18,82], likely through the direct inhibition of mitochondrial complexes I and II. Additionally, SV exerts pleiotropic effects that contribute to ROS production via alternative mechanisms [58,83]. Li et al. [58] demonstrated that SV not only induces ROS production but also upregulates the expression of the antioxidant enzyme superoxide dismutase (SOD). In contrast, LN has been shown to significantly reduce SOD activity in various cancer cells [82,84]. These findings suggest that LN amplifies ROS production compared to SV alone and enhances the pro-oxidative effects of SV by inhibiting SOD activity. This dual action likely accounts for the substantial increase in ROS production observed with NLC/LN/SV.

To gain insight into the role of ROS in the cytotoxic activity of NLC/LN/SV, A549 cells were incubated with NLC/LN/SV (5, 10, or 20 μM SV) for 24 h in the absence or presence of an antioxidant compound (NAC, 5 mM), and cell viability was measured. As shown in Figure 9C, NAC partially rescued cell viability from 23.5 to 45.6% in cells exposed to NLC/LN/SV at 10 μM SV (*p* < 0.01). We conclude that other antiproliferative mechanisms independent of ROS are likely involved at lower concentrations of NLC/LN/SV (and lower levels of ROS), since NAC was not able to prevent such cell growth inhibition.

#### 3.7.2. Effects of NLC/LN/SV on Mitochondrial Membrane Potential

**Figure 10:**
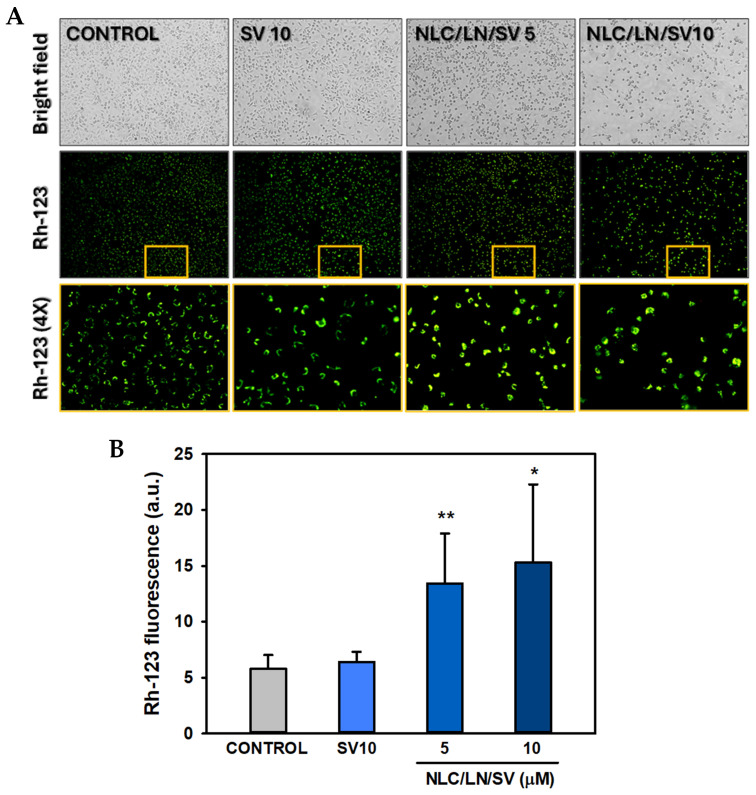
Effects of NLC/LN/SV on mitochondrial membrane potential in A549 cells. (**A**) Cells exposed to ethanol 0.2% (control), SV 10 µM, or NLC/LN/SV at 5 µM and 10 µM SV for 24 h, followed by staining with rhodamine-123 (Rh-123) (100×). Cells were analyzed and imaged using a fluorescence microscope. The region inside the yellow box is magnified 4× to enhance visualization of the green fluorescence signal. (**B**) Quantitative analysis of Rh-123 green fluorescence in A549 cells. Fluorescence intensity was quantified employing ImageJ 1.53 k (a.u. = arbitrary units). Data are presented as mean ± SD (*n* = 6). * *p* < 0.05; ** *p* < 0.01 vs. control.

This feature was evaluated by staining with Rh-123, whose accumulation in mitochondria is dependent on the membrane potential (Figure 10A). Mitochondria are a primary source of ROS, and excessive ROS generation may produce mitochondrial dysfunction, thus leading to membrane depolarization and, finally, triggering apoptosis [85]. On the other hand, various reports indicated that SV induced hyperpolarization of MMP and oxidative stress in cultured cells in conditions comparable to those evaluated here (10 µM SV, 24 h), leading to cell cycle arrest and apoptosis [86,87]. Here, we observed that free SV did not alter the MMP in A549 cells, whereas NLC/LN/SV (5 and 10 µM SV) significantly induced an increase in Rh-123 fluorescence of nearly 2 or 2.5-fold. Traditionally, the intrinsic apoptotic pathway was associated with the loss of MMP. Nevertheless, more recent studies have shown an increase in MMP during apoptosis initiation triggered by various stressors, including acrolein, cisplatin, staurosporine, or peroxide hydrogen, among others [88]. Moreover, increased levels of ROS induced the hyperpolarization of the MM [88].

Our results suggest that mitochondrial hyperpolarization may be involved in the NLC/LN/SV anticancer effects; however, further studies are required to shed light on this issue.

#### 3.7.3. NLC/LN/SV Promotes Cell Cycle Arrest and Cell Death

**Figure 11:**
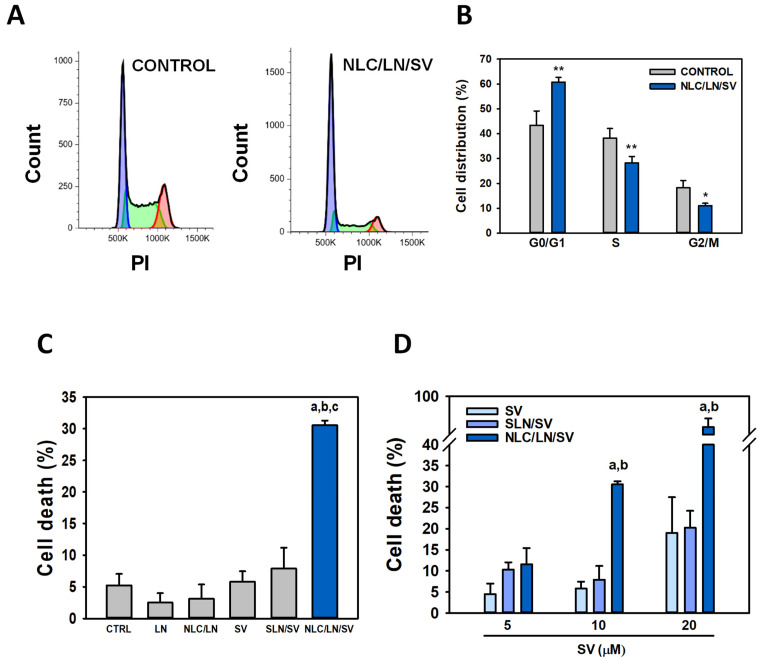
NLC/LN/SV induces G0/G1 cell cycle arrest and cell death. A549 cells were incubated with ethanol 0.2% (control) or NLC/LN/SV (10 µM SV) for 24 h. The cell cycle analysis was carried out by flow cytometry (**A**) and quantified (**B**). * *p* < 0.05; ** *p* < 0.01. (**C**) Cells were incubated with ethanol 0.2% (ctrl), free (SV) or encapsulated (SLN/SV) simvastatin (10 µM), free (LN) or encapsulated (NLC/LN) linalool (900 µM), or NLC/LN/SV for 24 h, and cell death was determined through trypan blue staining. (a) *p* < 0.001 vs. control; (b) *p* < 0.001 vs. free SV, (c) *p* < 0.001 vs. SLN/SV. (**D**) Cells were exposed to increasing concentrations of SV, SLN/SV, and NLC/LN/SV for 24 h, and cell death was analyzed by trypan blue staining. (a) *p* < 0.05 vs. free SV, (b) *p* < 0.05 vs. SLN/SV. Data are presented as the mean ± SD (*n* = 4).

Cell cycle arrest is regarded as one of the primary reasons for the inhibition of cell growth. SV, as well as different monoterpenes including LN, were shown to induce cell cycle arrest. We previously showed that free SV, free LN, and their combination arrested A549 cells in G0/G1 phase [19]. Here, we evaluated cell cycle progression by flow cytometry (Figure 11A). As shown in Figure 11B, NLC/LN/SV-treated cells exhibited a significant G0/G1 phase arrest, increasing from 43.3% in control cells to 60.8% (*p* < 0.01). Concurrently, a notable reduction in the S phase occurred, decreasing from 38.2% to 28.2% (*p* < 0.01), along with a decrease in the G2/M phase from 18.2% to 11.1% (*p* < 0.05).

To explore whether cell death accompanied cell cycle arrest as an anticancer mechanism mediated by NLC/LN/SV, A549 cells were first exposed to free LN, free SV, NLC/LN, SLN/SV, or NLC/LN/SV (10 µM SV, 900 µM LN in all cases) for 24 h, and cell death was then evaluated by the trypan blue exclusion method. Figure 11C shows that none of the individual components, encapsulated or in their free form, significantly promoted A549 cell death. On the other hand, NLC/LN/SV increased cell death from 5.2% (control cells) to 30.5% (*p* < 0.001). Finally, we analyzed the concentration-dependent effect of NLC/LN/SV, as well as free SV and SLN/SV, in the range of 5–20 µM of SV. As observed in Figure 11D, no statistical differences were observed for both free SV and SLN/SV compared to control cells, even at the maximal concentrations (20 µM SV), whereas NLC/LN/SV significantly induced A549 cell death starting from 30% at 10 µM SV and reaching 90% at 20 µM SV (*p* < 0.001).

Taken altogether, the results from cell viability, cell cycle, and cell death analyses suggest that NLC/LN/SV exerts cytostatic effects at low concentrations (5 µM), followed by a cytotoxic activity at moderate concentration (10 µM), which is maximal at the highest concentration assayed (20 µM).

#### 3.7.4. NLC/LN/SV Inhibits A549 Cell Migration

**Figure 12:**
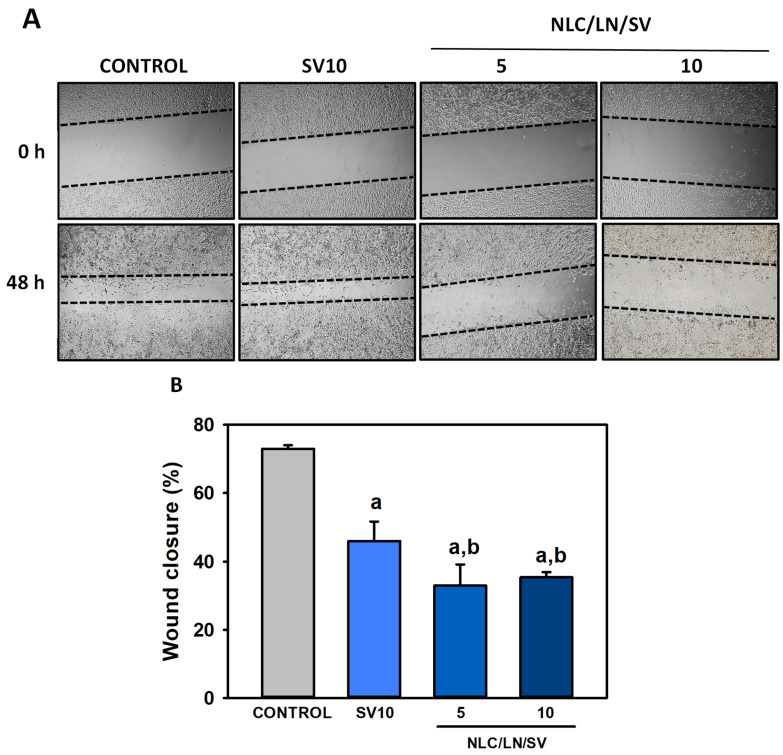
Encapsulation of SV enhances inhibition of cell migration. A549 cells were exposed to ethanol 0.2% (vehicle control), SV 10 µM, and NLC/LN/SV (5 and 10 µM), and the wound-healing assay was carried out. (**A**) Images representing the samples at 0 and 48 h (40×). (**B**) Analysis of wound closure in quantitative terms. Data are shown as mean ± SD (*n* = 4). (a) *p* < 0.05 compared to control; (b) *p* < 0.05 compared to free SV.

To further explore the anti-cancer potential of NLC/LN/SV in lung cancer cells, its effects on cell migration were evaluated through the wound healing assay (Figure 12A). Cell migration is a hallmark of cancer invasion and metastasis. This intricate biological process enables cancer cells to invade surrounding tissues and disseminate to various regions of the body, thereby facilitating metastases. Indeed, lung cancer is among those that most commonly metastasize [89]. Therefore, therapeutic approaches that integrate both cytotoxic and anti-metastatic characteristics are promising options for treating lung cancer. We found that free SV (10 µM) significantly reduced cell migration from 73.0% in control cells to 46.0% (*p* < 0.05) after 48 h (Figure 12B), consistent with previous reports [90]. Additionally, NLC/LN/SV exacerbated the antimigratory effects of free SV, even from lower concentrations, reducing cell migration to 33.0 and 35.3% at 5 and 10 µM SV, respectively (*p* < 0.05 vs. free SV in both cases). Cell migration and invasion are closely linked to the activity of Rho GTPases, which regulate the proliferative and invasive potential of various tumor cells, including lung cancer cells. Inhibition of RhoA prenylation in the mevalonate pathway by HMGCR inhibitors, such as SV, reduces the active Rho-GTP form, significantly suppressing cancer cell invasion and migration [91]. Moreover, SV has been shown to downregulate CD44 expression, a key regulator of lung cancer cell migration and invasion [92]. On the other hand, LN also inhibits protein prenylation, albeit through mechanisms distinct from SV [18,48] in addition to other recently described antimigratory mechanisms [93,94].

Our findings suggest that encapsulating SV within NLC/LN/SV enhances its antimigratory activity, probably as a consequence of LN and SV inhibiting cell migration at different levels.

## 4. Conclusions

To the best of our knowledge, this is the first report demonstrating the encapsulation of repurposed simvastatin into a bioactive NLC formulated with natural isoprenoids. Our findings revealed that NLC/LN/SV exhibited the highest cytotoxicity among the three phytoactive NLC formulations evaluated against A549 lung cancer cells. The co-encapsulation of LN and SV in a single NLC system significantly enhanced the cytotoxicity versus free or individually encapsulated drugs, achieving high encapsulation efficiency with monodispersed, stable nanoparticles of ~95 nm diameter. SAXS/WAXS studies revealed that LN altered the crystallographic architecture of the NLC, at both the core and the interface level, whereas SV inclusion did not affect matrix polymorphs. Sustained, controlled SV release was observed, reducing premature release risks in physiological conditions compared to SLN/SV.

NLC/LN/SV demonstrated high biocompatibility, showing no hemolysis and selective cytotoxicity against cancer cells over normal cells. In vitro studies revealed the formation of a thin protein corona around the NLC/LN/SV and a preference for fibrinogen interaction. NLC/LN/SV was efficiently incorporated by A549 cells, inducing ROS production, mitochondrial hyperpolarization, G0/G1 cell cycle arrest, and dose-dependent cell death, proving both cytostatic and cytotoxic anticancer effects. Finally, encapsulation of SV within NLC/LN/SV enhanced the inhibition of A549 cell migration.

Overall, NLC/LN/SV emerges as a promising anticancer delivery system for repurposing simvastatin to improve lung cancer therapy.

## Figures and Tables

**Table 1:** Composition of the different lipid nanoparticles.

Formulation	Lipid Phase			Aqueous Phase (20 mL)
	MM (mg)	Oil (μL)	SV (mg) *	Poloxamer 188 (p/v)
SLN	400	---	---	3.0%
NLC	400	500 (CRD)	---	3.0%
SLN/SV	400	---	12.6	3.0%
NLC/SV	400	500 (CRD)	12.6	3.0%
NLC/LN	400	500 (LN)	---	3.0%
NLC/LM/SV	400	500 (LM)	12.6	3.0%
NLC/CN/SV	400	500 (CN)	12.6	3.0%
NLC/LN/SV	400	500 (LN)	12.6	3.0%

* To obtain a formulation containing SV 1.5 mM.

**Table 2:** Mean, PDI, and Z-pot determined by DLS.

Formulation	Mean (nm)	PDI	Z-pot (mV)
SLN	132.2 ± 1.4	0.24 ± 0.01	−13.2 ± 0.6
NLC	144.0 ± 2.6	0.17 ± 0.02	−4.5 ± 0.7
SLN/SV	130.9 ± 1.5	0.25 ± 0.01	−16.3 ± 0.8
NLC/SV	142.4 ± 4.2	0.18 ± 0.01	−10.7 ± 0.3
NLC/LM/SV	120.4 ± 3.6	0.22 ± 0.01	−11.4 ± 0.7
NLC/CN/SV	142.8 ± 6.0	0.23 ± 0.01	−6.5 ± 0.6
NLC/LN/SV	94.2 ± 0.9	0.24 ± 0.02	−13.7 ± 0.9

**Table 3:** Lamellar parameters obtained from the 1D correlation curve.

Sample	Long Period (nm)	Amorphous Thickness (nm)	Crystalline Thickness (nm)	PDI	Local Crystallinity
P188 *	12.37 ± 0.01	4.42 ± 0.02	7.95 ± 0.02	0.44 ± 0.05	0.64 ± 0.06
SLN	12.37 ± 0.01	4.98 ± 0.05	7.39 ± 0.05	0.46 ± 0.08	0.59 ± 0.08
SLN/SV	13.21 ± 0.01	4.40 ± 0.07	8.81 ± 0.07	0.42 ± 0.08	0.67 ± 0.09
NLC/LN/SV	11.37 ± 0.01	4.78 ± 0.09	6.58 ± 0.09	0.51 ± 0.09	0.58 ± 0.09

* From previous work [32].

## Data Availability

The data that support the findings of this study are available from the corresponding authors, B.R.-K. and G.A.I., upon request.

## Supplementary Materials

The following supporting information can be downloaded at: https://www.mdpi.com/article/10.3390/pharmaceutics17020255/s1, Figure S1: Cytotoxicity of the vehicle (ethanol 0.2%), NLC/LN, NLC/LM, and NLC/CN on lung cancer A549 cells.
