# Supplementary material for: Phytoactive-Loaded Lipid Nanocarriers for Simvastatin Delivery: A Drug Repositioning Strategy Against Lung Cancer
Source: Pharmaceutics. 2025 Feb 14;17(2):255. doi: 10.3390/pharmaceutics17020255 (PMC11858925; doi:10.3390/pharmaceutics17020255)
Supplement: Supplementary file 1 [file pharmaceutics-17-00255-s001.zip › pharmaceutics-3367527-supplementary.pdf]

SUPPLEMENTARY MATERIALS

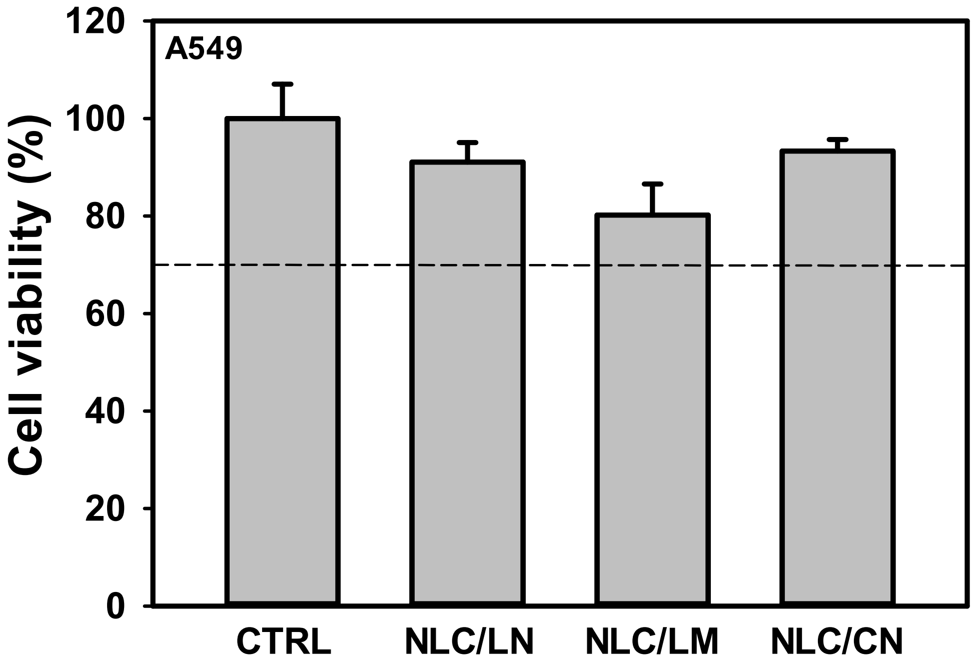

Figure S1. Cytotoxicity of the vehicle (ethanol 0.2%), NLC/LN, NLC/LM, and NLC/CN on lung cancer A549 cells. Cells were exposed to three different monoterpenes encapsulated into lipid nanoparticles at 2.5% v/v (NLC/LN, NLC/LM, and NLC/CN). Results are expressed as the mean ± SD (n = 8). The dotted line indicates 70% viability; below that, the formulation could be considered cytotoxic.
